# Robust ranks of true associations in genome-wide case-control association studies

**DOI:** 10.1186/1753-6561-1-s1-s165

**Published:** 2007-12-18

**Authors:** Gang Zheng, Jungnam Joo, Jing-Ping Lin, Mario Stylianou, Myron A Waclawiw, Nancy L Geller

**Affiliations:** 1 Office of Biostatistics Research, National Heart, Lung and Blood Institute, 6701 Rockledge Drive, MSC 7938, Bethesda, Maryland 20892 USA

## Abstract

In whole-genome association studies, at the first stage, all markers are tested for association and their test statistics or *p*-values are ranked. At the second stage, some most significant markers are further analyzed by more powerful statistical methods. This helps reduce the number of hypotheses to be corrected for in multiple testing. Ranks of true associations in genome-wide scans using a single test statistic have been studied. In a case-control design for association, the trend test has been proposed. However, three different trend tests, optimal for the recessive, additive, and dominant models, respectively, are available for each marker. Because the true genetic model is unknown, we rank markers based on multiple test statistics or test statistics robust to model mis-specification. We studied this problem with application to Problem 3 of Genetic Analysis Workshop 15. An independent simulation study was also conducted to further evaluate the proposed procedure.

## Background

For a large genetic study, a two-stage analysis is often employed. At the first stage, each marker is tested for association with a disease. The *p*-values of all markers are ranked. Then some of the most significant markers are analyzed in the second stage. This two-stage analysis reduces the number of hypotheses to be tested in the second stage. Hence, it enhances the power to identify true marker susceptibility to the disease. However, it is important to know how many of the most significant markers one should study in the second stage so that the probability that one or several true markers will be studied in the second stage is greater than a given value. On the other hand, when a given number of the most significant markers is selected, it is important to know the probability that this list of markers would contain one or more true markers. A small list of the most significant markers may not contain any true markers at all, which leads to spurious associations or negative findings in the second stage.

Zaykin and Zhivotovsky [1] used *p*-values of a single test statistic to rank markers. In a case-control study for complex diseases, three trend tests can be applied under the recessive, additive, and dominant models. Because the genetic model of the marker is uncertain, ranking the markers with a single test statistic may not be robust when another genetic model is correct. Using the first simulated data set of Problem 3 from Genetic Analysis Workshop (GAW) 15, we study robust ranking when the underlying genetic model is unknown and examine whether robust test statistics would lead to robust rankings of about 10 K single-nucleotide polymorphisms (SNPs). The properties of the proposed robust ranking procedures are then further examined by an independent simulation study.

## Methods

### Notation and model

Consider a SNP with alleles *D *and *d *and frequencies *p *and *q *= 1 - *p*, respectively. In a case-control design, *r *cases and *s *controls are independently sampled from a population. The genotype counts of three genotypes *G*_0 _= *dd*, *G*_1 _= *Dd*, and *G*_2 _= *DD *are denoted as (*r*_0_, *r*_1_, *r*_2_) in cases and (*s*_0_, *s*_1_, *s*_2_) in controls, which follow multinomial distributions *mul*(*r*: *p*_0_, *p*_1_, *p*_2_) and *mul*(*s*: *q*_0_, *q*_1_, *q*_2_), respectively. Denote the disease prevalence as *k *and penetrances as *f*_*i *_= P(case|*G*_*i*_) for *i *= 0, 1, 2. By the Bayes Theorem, *p*_*i *_= *g*_*i*_*f*_*i*_/*k *and *q*_*i *_= *g*_*i*_(1 - *f*_*i*_)/(1 - *k*), where *g*_*i *_= P(*G*_*i*_). Without loss of generality, assume that *D *has high risk. Then the null hypothesis of no association can be stated as *H*_0_: *f*_0 _= *f*_1 _= *f*_2 _= *k*. The alternative hypothesis is *H*_1_: *f*_0 _≤ *f*_1 _≤ *f*_2 _with at least one inequality. The genotype relative risks (GRRs) are defined as *λ*_1 _= *f*_1_/*f*_0 _and *λ*_2 _= *f*_2_/*f*_0_. The recessive, additive, and dominant models are referred to as *λ*_1 _= 1, *λ*_1 _= (1 + *λ*_2_)/2, and *λ*_1 _= *λ*_2_, respectively [2-4].

### Trend tests and robust tests

To test association using case-control data, the Cochran-Armitage trend test (CATT) has been proposed [2-4], which can be written as

$$Z_{x}=\frac{n^{1/2}\sum_{i=0}^{2}x_{i}\left(sr_{i}-rs_{i}\right)}{{\left[rsn\left\{n\sum_{i=0}^{2}x_{i}^{2}n_{i}-{\left(\sum_{i=0}^{2}x_{i}n_{i}\right)}^{2}\right\}\right]}^{1/2}},$$

where (*x*_0_, *x*_1_, *x*_2_) = (0, *x*, 1) and 0 ≤ *x *≤ 1. Given *x*, *Z*_*x *_follows asymptotically *N*(0,1). The choice of *x *is 0, 1/2, and 1 for the recessive, additive/multiplicative, and dominant models, respectively [5]. In practice, however, the true genetic model is unknown. Hence the robust tests, maximin efficiency robust test (MERT) and maximum test (MAX), can be applied, which are given by MERT = (*Z*_0 _+ *Z*_1_)/{2(1 + *ρ*)}^1/2 ^and MAX = max(|*Z*_0_|, |*Z*_1/2_|, |*Z*_1_|), where *ρ *= [*n*_0_*n*_2_/{(*n*_0 _+ *n*_1_)(*n*_1 _+ *n*_2_)}]^1/2 ^[4]. Note that Pearson's association test can also be used. However, Zheng et al. [6] showed that the MAX is often more powerful than the Pearson chi-squared test for a case-control design. Comparison of MERT and MAX can be found in Freidlin et al. [7]. The MAX and MERT have also been applied to other designs for GAW14 [8,9].

### Ranking markers with multiple statistics

When the genetic model is unknown, the three CATTs (*Z*_0_, *Z*_1/2_, *Z*_2_) are calculated for each of *M *SNPs. Then the *p*-values of MERT and MAX can be obtained for ranking. However, computing the *p*-value of MAX needs extensive simulation. Thus, alternatively, the minimum of the *p*-values (min *p*) of the three CATTs can be used for ranking. Rather than ranking *M *SNPs based on any single CATT, we propose ranking the SNPs by the MERT and the minimum of the *p*-values. We expect that ranking SNPs based on this approach would be more robust compared to ranking by a single CATT when the ranks by the three CATTs are quite different.

## Results

### Application to GAW15

As an application, we consider the first simulated data set of Problem 3 from GAW15. A simulated data set was considered, as we knew that there were eight candidate genes. One of them at chromosome 6 with physical location 32,484,648 bp was simulated based on the *DRB1 *locus of the *HLA *gene. We selected four SNPs closest in physical distance to the eight known candidate genes as candidate SNPs. We examined the ranks of the 32 candidate SNPs among all 9187 SNPs. All 2000 unrelated controls were used. For the affected sib-pair (ASP) data, we selected an affected sib (case) with the first individual ID from each family. A total of 1500 unrelated cases were used. In the simulated data set, genotypes of all 9187 SNPs from 22 chromosomes were generated (no missing genotypes and no genotyping errors). All SNPs had minor allele frequency (MAF) greater than 1% and there were no monomorphisms. Because we considered the CATTs, Hardy-Weinberg equilibrium in the population was not required [2]. If any genotype count in cases or controls was 0, 0.5 was added to all genotype counts in cases and controls.

After Bonferroni correction for *Z*_0 _(*Z*_1/2 _and *Z*_1_), there were 5 (7 and 7) SNPs among the 32 candidate SNPs that had Bonferroni-corrected *p*-values less than 0.05. All three CATTs, the MERT, and the minimum of the *p*-values of the three CATTs were used to rank all 9187 SNPs. The ranks of the 32 candidate SNPs are reported in Table 1 by five different ranking methods. The results are summarized below: 1) in the candidate gene *DRB1 *of *HLA *(chromosome 6, location = 32,484,648), four of the six most significant candidate SNPs are in this region. This implies that when the sample size and a genetic effect are large, a strong candidate gene should contain several SNPs at the top of the list of most significant SNPs. 2) Using a single CATT to rank SNPs may not be robust, and using MERT or the minimum *p*-value is more robust. For example, the SNP (chromosome 6, location = 37,363,880) has rank of 6 using either *Z*_1/2 _or *Z*_1_, and 8172 when *Z*_0 _is used. But the ranks of this SNP by MERT and minimum *p*-value are 10 and 6, respectively. 3) When the ranks by the three CATTs are quite different, the ranks by the robust methods are usually in the middle. 4) With a sample size of 3500, some candidate SNPs have ranks larger than those of null SNPs. Thus, selecting only the most significant SNPs from the genome-wide scan for further analysis may exclude some true associations or candidate genes. This information is particularly important for cost-efficient two-stage design for genome-wide association studies (e.g., Skol et al. [10]) in which only a portion of samples will be genotyped in the first stage to select markers to be genotyped using the remaining samples.

**Table 1:** Ranks of candidate genes among 9187 SNPs across 22 chromosomes based on five ranking methods, sorted by chromosome and location

			Rank				
			
Chr	Location (bp)	Diff^a^	*Z*_0_	*Z*_1/2_	*Z*_1_	min *p*	MERT
6	32447149	37 kb	4	4	4	3	4
6	32499465	14 kb	2	2	2	1	2
6	32521277	36 kb	3	3	3	2	3
6	32772203	387 kb	5	5	5	4	5
6	36900959	330 kb	966	1190	2028	1881	647
6	37363880	130 kb	8172	6	6	6	10
6	37539191	300 kb	6359	1430	464	931	2897
6	37657759	423 kb	968	1341	4671	1884	1414
8	140606402	3.2 mb	3012	4237	5775	5167	3328
8	140676097	3.1 mb	8391	7443	7097	8726	7382
8	140679773	3.1 mb	7936	7288	7096	8727	7225
8	142073109	1.7 mb	8918	6991	6588	8459	7407
9	25996861	262 kb	2921	4074	6290	5039	3556
9	26089466	169 kb	2179	9009	4702	3930	6948
9	26484252	225 kb	2374	2254	4205	3889	2291
9	26521692	262 kb	2909	2113	2819	3677	1947
9	27418665	118 kb	3667	3963	6458	5915	4070
9	27505967	31 kb	6228	7286	8222	8279	7989
9	27697461	160 kb	5582	7177	5317	7490	8915
9	27697600	160 kb	5195	4841	3323	5329	7532
11	110204257	30 kb	1	1	1	5	1
11	110259778	24 kb	3492	3162	4276	5125	2930
11	110264385	29 kb	271	222	857	419	186
11	110322303	87 kb	6840	3492	1930	3411	3030
16	12527182	9 kb	7729	4194	4148	6328	4884
16	12577812	60 kb	4288	5913	4696	6589	8924
16	12618035	100 kb	6212	7783	8356	8266	6771
16	12783679	266 kb	5824	4802	5334	7101	4733
18	65844474	225 kb	6522	4959	5282	7254	4864
18	66045171	24 kb	7063	8720	9182	8750	7913
18	66048927	20 kb	15	15	15	15	13
18	66230498	160 kb	5441	6135	6872	7732	5409

^a^Diff is the distance to the closest candidate gene

### An independent simulation study

To further study the properties of the robust ranking procedures, we conducted an independent simulation study. We simulated a case-control genome-wide association study of 100,000 SNPs with 500 cases and 500 controls. For illustration, we simply assumed that all SNPs were in linkage equilibrium, among which 9 SNPs were associated with a disease (3 SNPs had recessive, additive, and dominant modes of inheritance, respectively). The MAFs for the recessive, additive, and dominant SNPs were set at 0.3. MAFs for other null SNPs were generated from a uniform distribution (0, 1). The GRRs for each genetic model were specified. We repeated simulations of 100 K SNPs ten times and the average ranks for the 9 candidate SNPs were obtained and reported in Table 2. As in Table 1, min *p *and MERT are more robust than a single trend test (*Z*_0_, *Z*_1/2_, or *Z*_1_) for genome-wide scans. For example, for SNPs 3, 6, and 9 (having the greatest GRRs for each genetic model), the ranks of min *p *and MERT across three genetic models are all on the list of top 100 most significant SNPs, but are not if any single trend test is used.

**Table 2:** Average ranks of nine SNPs with true association in ten replicates in a genome-wide association study with 100 K SNPs

			Rank				
			
Model	SNPs	*λ*_2_	*Z*_0_	*Z*_1/2_	*Z*_1_	min *p*	MERT
Recessive	1	1.5	17582.8	15273.2	33593.4	16675.5	14511.4
Recessive	2	2.0	645.5	2591.3	21714.1	1331.2	1476.4
Recessive	3	2.5	1.5	385.9	19531.0	4.2	82.6
Additive	4	1.5	10106.3	5501.4	12420.2	6265.1	4808.4
Additive	5	2.0	5054.7	49.9	65.7	91.0	78.9
Additive	6	2.5	440.6	2.6	3.5	3.3	2.5
Dominant	7	1.5	30772.7	3510.1	3118.1	4245.9	4980.7
Dominant	8	2.0	11644.0	6.8	3.1	4.1	19.0
Dominant	9	2.5	6364.8	1.3	1.0	1.2	1.8

## Conclusion

In this article, we studied the robust properties of ranks of true associations in genome-wide scans. In some situations, ranking markers by a single trend test may not be robust, in particular, when the true genetic model is unknown. Using robust methods, such as min *p *and MERT, to rank markers may lead to higher power when the ranks by three CATTs are quite different. The results showed that they are particularly useful in ensuring that recessive effects are not missed. While min *p *and MERT improve the univariate approach to the first stage of gene discovery, simulated data shows that some SNPs are not found via these univariate methods.

## Competing interests

The author(s) declare that they have no competing interests.
